# Case Report: Christianson Syndrome Caused by *SLC9A6* Mutation: From Case to Genotype-Phenotype Analysis

**DOI:** 10.3389/fgene.2021.783841

**Published:** 2021-12-20

**Authors:** Yueyun Lan, Sheng Yi, Mengting Li, Jinqiu Wang, Qi Yang, Shang Yi, Fei Chen, Limei Huang, Yiyan Ruan, Yiping Shen, Jingsi Luo, Zailong Qin

**Affiliations:** ^1^ Guangxi Key Laboratory of Reproductive Health and Birth Defects Prevention, Guangxi Key Laboratory of Precision Medicine for Genetic Diseases, Guangxi Key Laboratory of Birth Defects and Stem Cell Biobank, Guangxi Key Laboratory of Birth Defects Research and Prevention, Maternal and Child Health Hospital of Guangxi Zhuang Autonomous Region, Nanning, China; ^2^ Genetic and Metabolic Central Laboratory, Guangxi Birth Defects Research and Prevention Institute, Nanning, China; ^3^ The Third Affiliated Hospital of Guangzhou Medical University, Guangzhou, China; ^4^ Pediatrics Department, Maternal and Child Health Hospital of Guangxi Zhuang Autonomous Region, Nanning, China; ^5^ Department of Genetics, Harvard Medical School, Boston, MA, United States

**Keywords:** christianson syndrome, epilepsy, SLC9A6 gene, Na + /H + exchanger 6, electroencephalography, developmental delay

## Abstract

Christianson syndrome (CS) is an X-linked neurodevelopmental syndrome characterized by microcephaly, epilepsy, ataxia, and severe generalized developmental delay. Pathogenic mutations in the *SLC9A6* gene, which encodes the Na^+^/H^+^ exchanger protein member 6 (NHE6), are associated with CS and autism spectrum disorder in males. In this study, whole exome sequencing (WES) and Sanger sequencing revealed a novel *de novo* frameshift variant c.1548_1549insT of *SLC9A6* in a 14-month-old boy with early-onset seizures. According to The American College of Medical Genetics and Genomics (ACMG)/the Association for Molecular Pathology (AMP) guidelines, the variant was classified as pathogenic. The proband presented with several core symptoms of typical epilepsy, including microcephaly, motor delay, distal muscle weakness, micrognathia, occasional unprovoked laughter, swallowing and speech difficulties. Electroencephalography (EEG) showed spikes-slow waves in frontal pole, frontal, anterior temporal and frontal midline point areas. Gesell development schedules (GDS) indicated generalized developmental delay. We also summarized all the reported variants and analyzed the correlation of genotype and phenotype of CS. Our study extends the mutation spectrum of the *SLC9A6* gene, and it might imply that the phenotypes of CS are not correlated with *SLC9A6* genotypes.

## Introduction

Christianson syndrome (CS) is induced by sudden abnormal discharge of brain neurons, and is characterized by recurrent seizures due to hypersynchronous discharge (Liu et al., 2018). Symptoms of CS often begin in childhood, with a complex clinical manifestation that is associated with social and learning disabilities. Moreover, severe epilepsy comprises a group of devastating neurological disorders characterized by frequent epileptic seizures associated with developmental delay or regression (McTague et al., 2016). Each type of epilepsy has specific clinical manifestations and distinct underlying pathophysiologic or genetic mechanisms. It is believed that identifying the underlying cause of epilepsy or specific electroclinical syndrome is central to understand the natural history and optimal treatment of the condition (Wirrell, 2016).

To date, multiple genes including Na^+^/H^+^ exchanger protein 6 (NHE6, also known as *SLC9A6*) have been linked to CS (McTague et al., 2016). NHE6 plays a role in both excitatory presynaptic and postsynaptic functions (Deane et al., 2013). Additionally, NHE6 is highly expressed in the brain, and is thought to participate in the targeting of intracellular vesicles and recycling of synaptic vesicles. Therefore, mutations leading loss of function in *SLC9A6* protein may damage the development of neurons (Mignot et al., 2013). Furthermore, *SLC9A6* has been identified as a pathogenic gene (Ikeda et al., 2020) and its pathogenic variations can lead to an Angelman-like syndrome (AS-like, known as CS) (Cardon et al., 2016), which is characterized by epilepsy, intellectual disability, microcephaly, ophthalmoplegia, craniofacial dysmorphism and progressive cerebellar atrophy (Padmanabha et al., 2017).

Childhood epilepsy, especially the seizures, is manifested in the context of brain development and can change over time. CS in children presents a wide range of treatment challenges that are unique to this age group. Therefore, it is important to distinguish a *de novo* mutation caused childhood epilepsy or hereditary childhood epilepsy, and effective therapies for different forms of epilepsy should be targeted to the individual child according to the type of seizures. In this study, we identified a novel and *de novo SLC9A6* frameshift mutation c.1548_1549insT in the patient with tonic-clonic seizures and generalized developmental delay by whole exome sequencing (WES). We also summarized the pathogenic variants reported previously and widen the spectrum of *SLC9A6* mutations.

## Patient and Methods

### Editorial Policies and Ethical Considerations

Written informed consent for participation in this study was collected from the family members of patients. The study was approved by the ethics committees of Maternal and Child Health Hospital of Guangxi Zhuang Autonomous Region.

### Relevant Physical Examination and Accessory Examinations

The patient was a 14-month-old boy who presented with convulsions for a month without apparent trigger. In order to clarify the etiology and clinical diagnosis, the patient underwent physical examination and clinical auxiliary examination during hospitalization, including rehabilitation training treatment, three usual medical practices, blood biochemistry, 24-hour ambulatory electroencephalogram, visual-auditory synchrony evoked potential, abdominal ultrasonography (US), the cerebrospinal fluid (CSF), routine electrocardiogram (ECG), thyroid function teats and genetic testing (WES and Sanger sequencing).

### Molecular Analysis

Genomic DNA was isolated from peripheral blood lymphocytes using LabAid DNA kit (Zeesan Biotech Co., Ltd, Xiamen, China). For WES, genomic DNA sample of the patient were captured to create a sequencing library by Agilent SureSelect Clinical Research Exome V2 Kit (Agilent Technologies, Santa Clara, CA), and the prepared libraries were sequenced on a HiSeq2500 (Illumina, San Diego, CA). Sequence alignment and variant calling against the reference human genome (GRCh37) were performed using Burrows-Wheeler Aligner (BWA) and the Genome Analysis Toolkit (GATK) (Van der Auwera et al., 2013). Copy number variants (CNV) analysis based on reads depth method was performed using an inhouse pipeline, and CNVs of significant interest were further visually inspected with the Integrative Genomics Viewer. CNVs and indels were annotated and prioritized by the TGex software (LifeMap Sciences, Alameda, CA). The variant pathogenicity was assessed according to American College of Medical Genetics and Genomics (ACMG)/Association for Molecular Pathology (AMP) guidelines (Richards et al., 2015). All operations were carried out according to the instructions of the manufacturer.

## Results

### Clinical Manifestations

**FIGURE 1:**
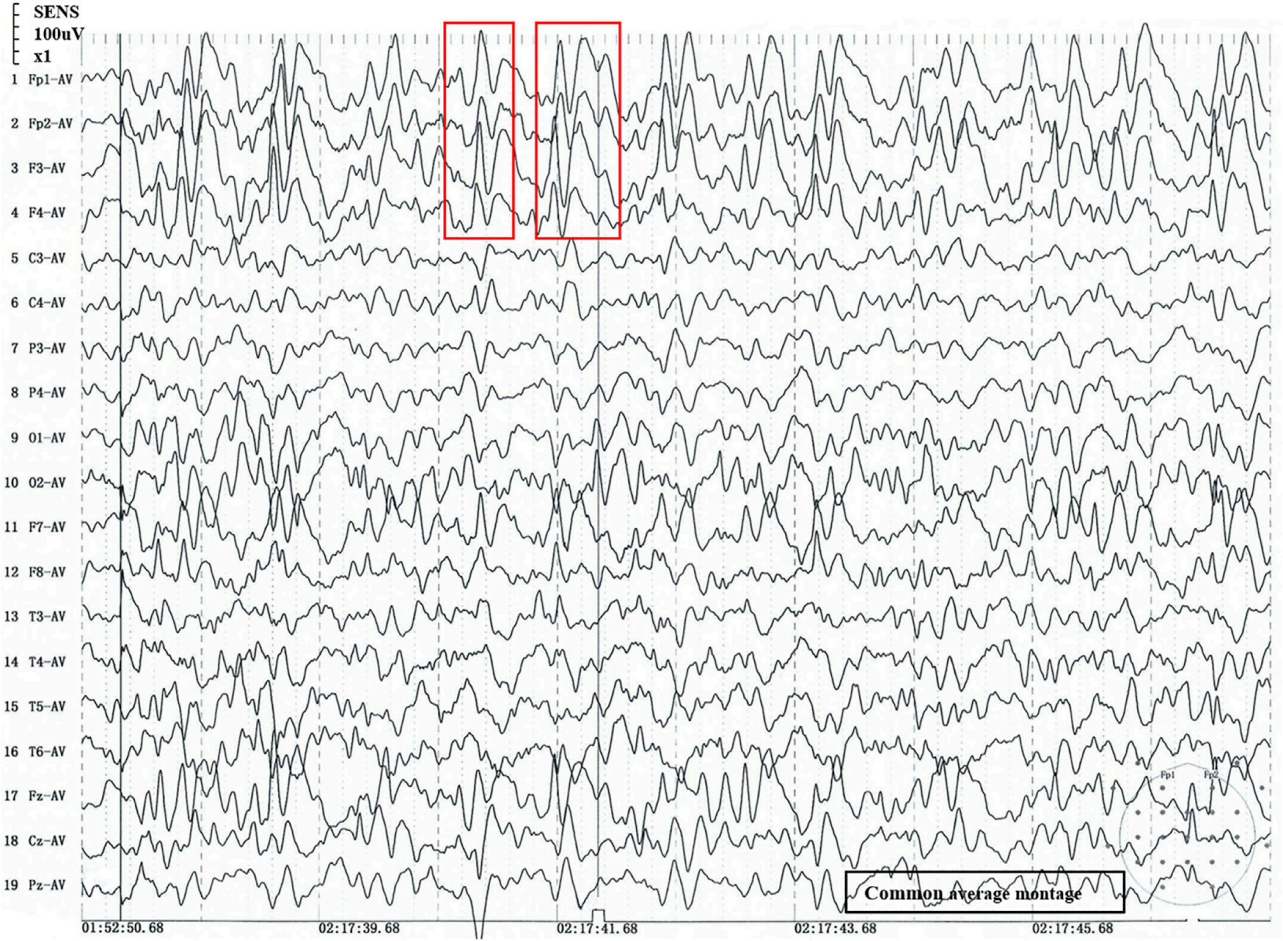
Sleep stage II EEG. 1) 1 to 19: Recording electrode. 2) Fp1-AV to Pz-AV: Average montage. 3) Fp1/Fp2: pre frontal lobe, F7/F8: inferior frontal lobe, F3/F4/Fz: frontal lobe, C3/C4/Cz: central lobe, T3/T4: temperal lobe, T5/T6: posterior temperal lobe, P3/P4/Pz: parietal lobe, O1/O2: occipital lobe; The number of the recording electrodes mentioned above is usually odd for the left and even for the right. 4) Red box: abnormal wave. 5) The time window is 2 s, and every 2 s in the signal is an epoch.

The patient was delivered to term with a weight of 3200 g at birth, did not undergo suffocation rescue when he was born, and had jaundice but did not receive phototherapy, adopt exclusive breastfeeding after birth and give supplementary food on time. The patient lagged behind his peers in growth and development. At the age of 5 months, he could roll over; at 6 months, he could hold his head up and at 9 months, he could sit up. At the age of 14 months, he could crawl and stand with support. However, tonic-clonic seizures began at 13 months of age and occurred several times a day. Examination revealed microcephaly (43cm, −3SD), motor delay, distal muscle weakness, micrognathia, convulsions, slender fingers, adduction of the thumbs and occasional unprovoked laughter. He also had swallowing and speech difficulties. We used Gesell development schedules (GDS) to assess the level of development. The GDS revealed the patient had delays in adaptation, large motor movement, fine motor movement, personal-social development (mild), and language (moderate). The patient was diagnosed with tonic-clonic seizures and generalized developmental delay. Therefore, it is suggested that the patient should be treated with rehabilitation training. Figure 1 showed the results of sleep EEG using common average montage, EEG demonstrated that 3.5–8 Hz diffuse wave as background activity, spike-slow waves discharged in frontal pole, frontal, anterior temporal and frontal midline point areas. Brain magnetic resonance imaging, brain-stem auditory evoked potential and visual evoked potential showed no abnormal findings.

#### Clinical Auxiliary Examination

Relevant data from the episode of care is shown in Supplementary Table S1: 1) Blood ammonia level [54.0 μmol/L, reference range 11–35 μmol/L]. 2) Routine blood test: red blood cell (RBC) [3.9×10^9^/L, reference range (4.3–5.8) ×10^12^/L], hemoglobin (Hb) [107 g/L, reference range 180–190 g/L]. 3) Lactic acid [9.41 mg/dL, reference range 12.00–16.00 mg/dL]. 4) Determination of serum immunoglobulin: immunoglobulin A (IgA) [0.30 g/L, reference range 0.70–3.30 g/L]. 5) Thyroid function: thyroxine (T4) [69.70 nmol/L, reference range 66.00–181.00 nmol/L]. 6) Routine CSF: serum sodium valproate concentration [41.14 μg/mL, reference range 50–100 μg/mL], herpes simplex virus type I, herpes simplex virus type II and CSF culture were negative. 7) Routine Conventional electrocardiogram (ECG) examination found that the patient had sinus tachycardia with irregularities. 8) 25-hydroxy vitamin D level, 25-hydroxy vitamin D3, blood gas analysis, ferritin, calcitonin, electrolytes, zinc, iron and Anti-Streptolysin O, stool routine test and abdominal ultrasound were normal. The results of other related testing items involved were normal.

### Molecular Genetic Analysis

**FIGURE 2:**
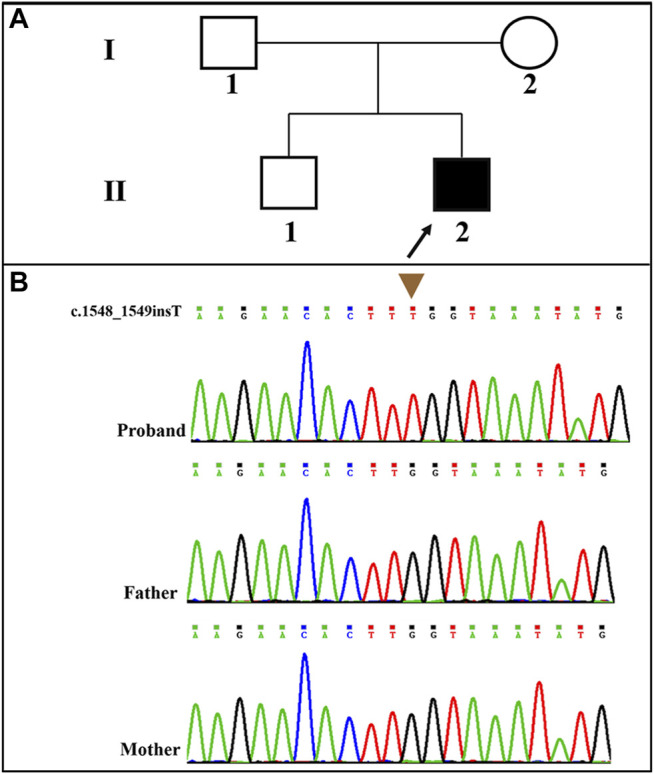
**(A)**: Pedigree of the proband; **(B)**: The patient carried a novel and *de novo* hemizygous *SLC9A6* mutation (NM_006359.2: c.1548_1549insT) that was confirmed by Sanger sequencing.

A novel frameshift mutation, c.1548_1549insT (p. Leu517fs*5), was identified in the *SLC9A6* gene (NM_006359.2) by WES analysis. This variant was absent in the Clinvar Database, the Single Nucleotide Polymorphism database and the Human Gene Mutation Database. The novel variant was validated by Sanger sequencing. The child’s family history was unremarkable. The pedigree of the family was shown in Figure 2A. *SLC9A6* mutation (c.1548_1549insT) was not detected in peripheral blood samples obtained from the parents (Figure 2B). The mutation was considered to be *de novo*. The paternity was confirmed by short tandem repeat markers. According to the ACMG/AMP guidelines, this novel and *de novo* frameshift variant was classified as a pathogenic variation (PVS1+PM2_supporting+PS2+PP4): a null variant (frameshift) was confirmed in the gene that loss of function is a known mechanism of disease (PVS1); a *de novo* variant (both maternity and paternity confirmed) in a patient with the disease and without family history (PS2); absent from controls (or at extremely low frequency if recessive) in the Exome Sequencing Project, 1,000 Genomes Project, Exome Aggregation Consortium or gnomAD databases (PM2).

### Clinical Diagnosis

The main clinical manifestations of patients with central nervous system infection are fever, convulsions, disturbance of consciousness and increased intracranial pressure. In addition, these patients had poor mental state and CSF was altered accordingly. Genetic metabolic diseases can lead to feeding difficulty, developmental delay, convulsions, specific facial features, electrolyte abnormalities, damage of important organs. Physical examination showed rash and hepatosplenomegaly. However, no obvious abnormalities were found in nervous system and skin examination of our patient. In view of this, it can be diagnosed that the convulsions of our patient were not caused by central nervous system infection or genetic metabolic diseases. The decrease of glucose content in CSF is the characteristic of glucose transporter type 1 deficiency syndrome, but the results of CSF examination in our patient were normal, so it can be ruled out that seizures are related to glucose transporter-1 deficiency syndrome. Electrolyte disorder, such as low calcium and low magnesium, can cause convulsions, but the diagnosis was not supported because our patient had no abnormal electrolyte after admission. To summarize, epilepsy with tonic-clonic seizure and generalized developmental delay of the patient was caused by *SLC9A6* mutation.

## Discussion

CS is a rare but increasingly diagnosed neurodevelopmental and degenerative form of X-linked intellectual disability disorder. NHE6 regulates recycling endosomal pH homeostasis and trafficking (Petitjean et al., 2020). The ion transporter is abundantly expressed in the central nervous system (CNS), which may explain why the neural phenotype of CS is so complex and diverse. Therefore, the identification of NHE6-dependent specific receptors helps to clarify the underlying mechanism of neuronal dysfunction in CS. Endosomal acidification as a result of a non-functional or absent NHE6 caused by *SLC9A6* gene mutation may affect the transport of vesicles containing AMPA receptors to and from the postsynaptic membrane (Shepherd and Huganir, 2007). Brain-derived neurotrophic factor (BDNF)/tropomyosin receptor kinase B (TrkB) neurotrophin signaling pathway are necessary for correct dendrite development in many neurons in the CNS (Barford et al., 2017). Coupled with accelerated degradation of TrkB, this over-acidification can lead to a disruption of endosomal BDNF/TrkB signaling, resulting in the failure of neuronal axons and dendritic branches (Fuster and Alexander, 2014).

In addition, microtubule-associated proteins (MAP) also play a significant role in neurodevelopment and growth. Tau protein is the main microtubule associated protein of mature neurons. The spatiotemporal expression of different tau isoforms is a characteristic during brain development (Avila et al., 2004), suggesting that the regulation of tau isoforms is important during the formation of the brain (Lee et al., 1989). Moreover, deposition of tau may be mediated by the interaction with the mutant *SLC9A6* protein (Garbern et al., 2010). After using a conservative genome-wide correction, it was found that the low expression of *SLC9A6* was significantly related to increased tau deposition (Pescosolido et al., 2019). The most obvious pathological event in several neurodegenerative diseases is the aggregation of tau subtypes into the filamentous content of neurons, resulting in hyperphosphorylation of tau proteins (Buée et al., 2000). The c.1548_1549insT pathogenic variant in the *SLC9A6* gene causes a frameshift starting at codon Leucine 517, which changes this amino acid to a proline residue, and creates a premature stop codon at position 5 of the new reading frame, denoted p.Leu517fs*5 which cause premature termination of NHE6 protein synthesis and may greatly reduce the stability of tau proteins. It can be further speculated that epilepsy and developmental delay in our patient may be due to the inability of the mutant *SLC9A6* to compensate in those tissues.

Mutations in *SLC9A6* seem to cause clinical symptoms that reflect developmental and progressive pathophysiology (Pescosolido et al., 2014). Slc9a6 knockout mouse models revealed that loss of functions of *SLC9A6* leads to altered endosomal-lysosomal function and cholesterol accumulation in some neuronal populations in a manner that is similarly toprimary lysosomal storage diseases (Sinajon et al., 2016). Moreover, Slc9a6^−/−^ mutant female mouse and Slc9a6–/0 mutant male mouse showed an epileptic phenotype and reduced seizure threshold (Gilfillan et al., 2008). One of the causes of cognitive and language impairment in children with *SLC9A6* mutations may be the failure of axonal and dendritic branching, leading to impaired neuronal connectivity (Roxrud et al., 2009).

**FIGURE 3:**
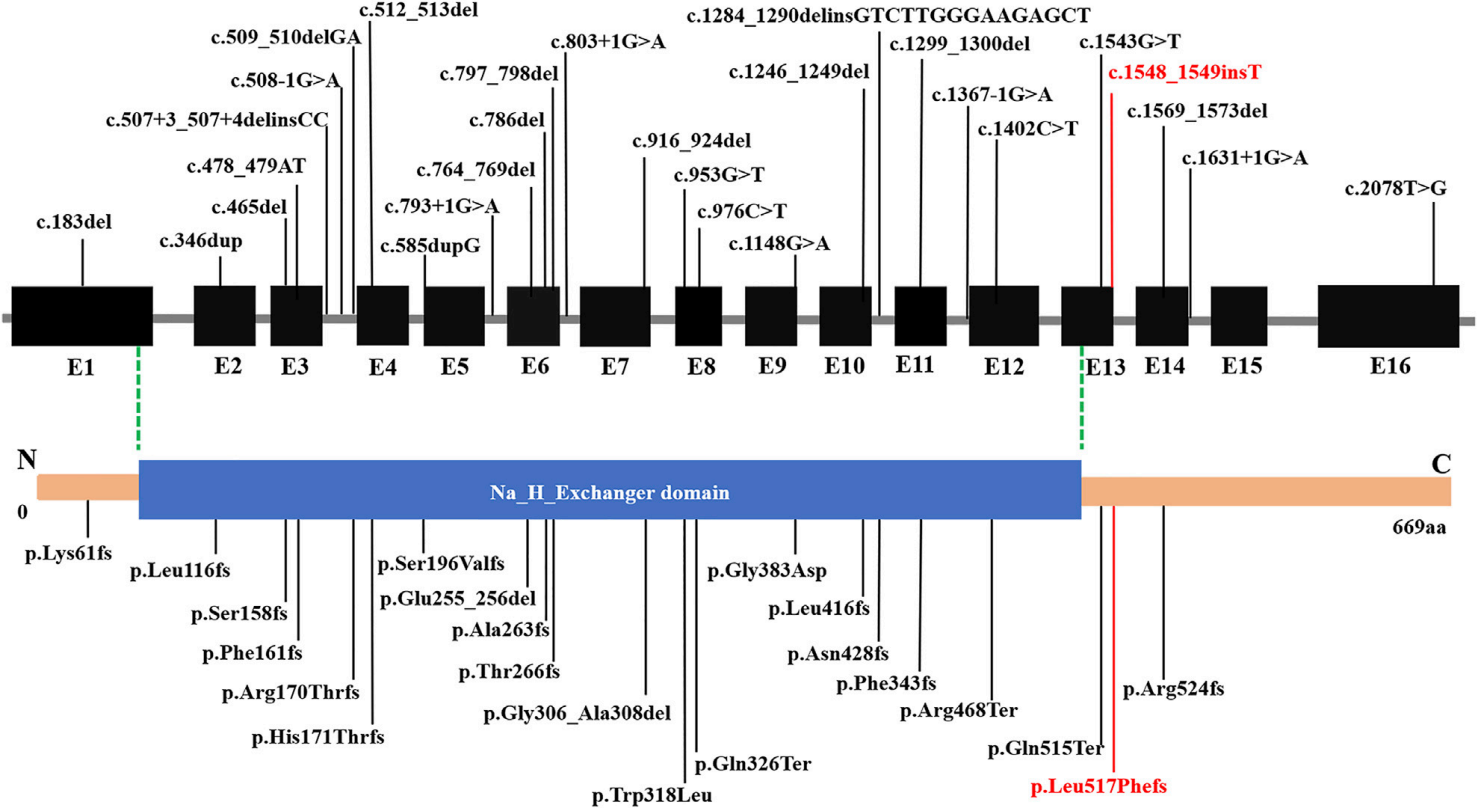
Structure of *SLC9A6* protein. The *SLC9A6* gene is composed of 669 amino acids and has only one domain. Predicted functional domains are shown together with the position of a novel *de novo* mutation in the report. *SLC9A6* conserved domain was predicted by NCBI Conserved Domain Database. The black fonts: Previously reported pathogenic mutations, the red fonts: pathogenic mutations reported in this paper.

Disruption of *SLC9A6* should be considered in male patients with a non-15q11-13-related AS phenotype, particularly when X-linked inheritance in the family is suspected. Moreover, CS and AS share some common clinical features, and part of the patients with AS-like phenotypes carried mutation in *SLC9A6* gene. Supplementary Table S2 summarizes the main clinical phenotypes of *SLC9A6* mutations. AS-like phenotype was observed in the related families described by case 1 and case 2. Developmental delay, microcephaly, seizures, language retardation were present in most patients. Intellectual disability, behavior disorder and ataxia are seen in several cases. The phenotype of patients in the table is consistent with clinical diagnosis, but genotype and phenotype correlations were confirmed to be negative. Subsequently, *SLC9A6* conserved domain was predicted (Figure 3). Total of 28 pathogenic variants were reported in ClinVar Database. However, 22 out of 28 (78.57%) pathogenic variants in *SLC9A6* located in predicted functional domains, which include frameshift mutations, missense mutations and splicing mutations. And CS was the most reported clinical phenotype in these variations. Although 6 variants (21.43%,6/28) located in the non-domain region, 5 of them with CS located in non-domain region. It might imply that there was no significant correlation between the type of *SLC9A6* gene variant and the clinical phenotype and protein domain.

Based on the information of *SLC9A6* gene variation and clinical phenotype included in ClinVar Database (SLC9A6 [gene]–ClinVar–NCBI (nih.gov)), we summarized the variants that were only caused by *SLC9A6* mutations (Supplementary Table S3): 1) All frameshift mutations (N = 13) of *SLC9A6* are pathogenic variations. The clinical outcomes of partial variation were related to CS and no phenotype was reported for the remaining variants. 2) Seven cases of single nucleotide variants (SNVs) were pathogenic and one case were likely pathogenic, which were also associated with CS. Most SNVs in *SLC9A6* were not pathogenic, but the phenotypes of most patients with SNVs were typical CS. The frameshift mutations seem to be more serious than the SNVs. Taken together, no matter the type of *SLC9A6* gene mutation is frameshift mutations or SNVs, the clinical phenotype mainly characterized by CS and developmental disorder. Moreover, the complexity of the neuronal arbors in CS patients was reduced regardless of the nature of the mutation and the deficiency may contribute to postnatal microcephaly in CS (Lizarraga and Ma, 2021). The great majority of CS patients with *SLC9A6* mutations appear to be result in loss of function of the relevant proteins, such as putatively protein-truncating due to early frameshift, nonsense or splicing mutations, while some missense or intra-frame deletions may be residual protein (Pescosolido et al., 2014). The most recent research indicates that most CS mutations lead to loss of protein through nonsense-mediated decay (NMD) mechanisms, the loss and expression of wild-type *SLC9A6* in cells with an *SLC9A6* nonsense mutation could rescue the neuronal arborization defect; But cells with recurrent missense mutation (e.g., c.1148G > A, p.G383D) in the *SLC9A6* gene that resulted in a residual, nonfunctional NHE6 protein could not be rescued (Lizarraga and Ma, 2021).

## Data Availability

The original contributions presented in the study are publicly available. This data can be found here: PRJNA770360.

## Supplementary Material

Supplementary Table S1 A timeline of the patient's hospitalization that relevant data from the episode of care.

Supplementary Table S2 The main clinical phenotypes of patients with *SLC9A6* mutations.

Supplementary Table S3 The variants previously reported are shown in the excel according to Clinvar Database. 1) P=pathogenic, LP=likely pathogenic, B=benign, LB=Likely benign, VUS=Uncertain significance; 2) NR=not record, CS=not specified; 3) The point mutations with Conflicting interpretations of pathogenicity are not listed here.
