# Supplementary material for: Case Report: Christianson Syndrome Caused by SLC9A6 Mutation: From Case to Genotype-Phenotype Analysis
Source: Front Genet. 2021 Dec 20;12:783841. doi: 10.3389/fgene.2021.783841 (PMC8721738; doi:10.3389/fgene.2021.783841)
Supplement: Supplementary file 3 [file Table1.docx]

Supplementary Table 1.

| Case | Main clinical symptoms | SLC9A6 mutation | Location | Disease status |
| --- | --- | --- | --- | --- |
| 1 | Developmental delay, seizures, ataxia, flexed arms,  hyperkineticbehavior, spontaneous laughter, frequent  smiling | c.764_769delAAAGTG (p.E255_S256del),  c.507þ1delGTAA (p.V144_R169del),  c.512_513delAT (p.H171fs), c.1402C>T (p.R468X), | Exon 3, 4, 5, 12 | X-Linked mental retardation, ataxia microcephaly, epilepsy |
| 2 | General development delayed, poor eye contact,  microcephaly,hyperkinetic behavior, absent speech | c.25G>T (p.A9S) | Exon 1 | Angelman-like syndrome |
| 3 | Language retardation, autism spectrum, seizures,  late-onset ataxia,dystonia | c.1012_1020del | Exon 8 | Mental retardation |
| 4 | Unprovoked laughter, myoclonic jerks, microcephaly,  developmental delay, intellectual disability,  behavioral abnormalities,language retardation | c.916C>T (p.Gln306X) | Exon 7 | retinitis pigmentosum,  CS |
| 5 | Intellectual disability, microcephaly, social dysfunction | c.526‐9_526‐5del | Exon 3 | CS |
| 6 | Microcephaly, global developmental delay, serzure | c.190G>T (p.E64X) | Exon 1 | CS |
| 7 | Elongated forehead, depressed nasal bridge,  central hypotonia, brisk muscle stretch reflexes,  bilateral Babinski’s sign | c.1657G>A (p.E559K) | Exon 16 | Intellectual disability,  epilepsy |
| 8 | Developmental delay, language retardation, microcephaly,trunk ataxia, intractable seizures, frequentsmiling | c.1141-8C>A | Exon 10 | CS |
| 9 | Motor and intellectual retardation, poor speech,  microcephaly,ataxic gait,truncal hypotonia,  hyperkinesis | c.1402C>T (p.Arg468*),  c.477_481del(p.Ile160Leufs*5) | E12  E3 | CS |
| (10)  The report | Tonic-clonic seizures, microcephaly,  developmental delay, language retardation,  difficulty swallowing, spontaneous laughter | c.1548_1549insT (p. Leu517fs*5) | Exon 13 | CS |
